# 3q26 Amplification Is an Effective Negative Triage Test for LSIL: A Historical Prospective Study

**DOI:** 10.1371/journal.pone.0039101

**Published:** 2012-07-06

**Authors:** Erica R. Heitmann, Kamani M. Lankachandra, Jeff Wall, George D. Harris, Hollie J. McKinney, G. Reza Jalali, Yogita Verma, Eric Kershnar, Michael W. Kilpatrick, Petros Tsipouras, Diane M. Harper

**Affiliations:** 1 Department of Biomedical and Health Informatics, University of Missouri-Kansas City School of Medicine, Kansas City, Missouri, United States of America; 2 Department of Obstetrics and Gynecology, University of Missouri-Kansas City School of Medicine, Kansas City, Missouri, United States of America; 3 Department of Pathology, University of Missouri-Kansas City School of Medicine, Kansas City, Missouri, United States of America; 4 Department of Community and Family Medicine, University of Missouri-Kansas City School of Medicine, Kansas City, Missouri, United States of America; 5 Center of Excellence, Women’s Health, University of Missouri-Kansas City School of Medicine, Kansas City, Missouri, United States of America; 6 Ikonisys, Inc., New Haven, Connecticut, United States of America; Enzo Life Sciences, Inc., United States of America

## Abstract

**Background:**

Women with low grade squamous intraepithelial lesions (LSIL) at cervical cancer screening are currently referred for further diagnostic work up despite 80% having no precancerous lesion. The primary purpose of this study is to measure the test characteristics of 3q26 chromosome gain (3q26 gain) as a host marker of carcinogenesis in women with LSIL. A negative triage test may allow these women to be followed by cytology alone without immediate referral to colposcopy.

**Methods and Findings:**

A historical prospective study was designed to measure 3q26 gain from the archived liquid cytology specimens diagnosed as LSIL among women attending colposcopy between 2007 and 2009. 3q26 gain was assessed on the index liquid sample; and sensitivity, specificity, positive predictive value (PPV) and negative predictive value (NPV) were measured at immediate triage and at 6–16 months after colposcopic biopsy. The sensitivity of 3q26 gain measured at immediate triage from automated and manually reviewed tests in 65 non-pregnant unique women was 70% (95% CI: 35, 93) with a NPV of 89% (95% CI: 78, 96). The sensitivity and NPV increased to 80% (95% CI: 28, 99) and 98% (95% CI: 87, 100), respectively, when only the automated method of detecting 3q26 gain was used.

**Conclusions:**

3q26 gain demonstrates high sensitivity and NPV as a negative triage test for women with LSIL, allowing possible guideline changes to routine surveillance instead of immediate colposcopy. Prospective studies are ongoing to establish the sensitivity, specificity, PPV and NPV of 3q26 gain for LSIL over time.

## Introduction

Human papillomavirus (HPV) and telomerase overexpression have been recognized as independent carcinogenic factors for which the Nobel Prize in Physiology or Medicine was awarded in 2008 and 2009, respectively. Harald zur Hausen defined the link between human papillomavirus (HPV) infection and cervical cancer; and Elizabeth Blackburn, Carol Greider and Jack Szostak detailed the function of telomeres and the enzyme telomerase. Cells that are becoming cancerous or are cancerous express telomerase over-abundantly, allowing gains in the chromosomes’ telomere length. Testing for gains in human chromosome length of HPV infected cells may identify a potential biomarker for cervical cancer screening.

The primary screening method for cervical cancer has been the morphologic characteristics of HPV infection in the epithelial cells of the uterine cervix. The Bethesda System, (TBS-2001) [1], [2] has replaced the original Papanicolaou classification system for cervical cytologic reporting in many parts of the world. The cervical intraepithelial neoplasia (CIN) grade 1, 2 and 3 nomenclature is reserved for histologic classification of morphologic evidence of HPV associated changes and, in general, corresponds to the cytologic diagnoses: low grade squamous intraepithelial lesion (LSIL) to CIN 1; and, high grade squamous intraepithelial lesion (HSIL) to CIN grades 2 and 3 [3]. The cytologic screening diagnosis of LSIL makes up 24–31% of all abnormal cytology reports [4], [5]. While LSIL is the most concordant diagnosis among cytopathologists [1], nearly 35% of all LSIL diagnoses are both over- and under-called with respect to inter-observer agreement [6]. In addition, nearly 20% of the LSIL cytology represents cervical intraepithelial neoplasia grade 2 or 3 (CIN 2/3) disease at colposcopy [7]–[11], not the histologic CIN 1 usually associated with LSIL. Inversely, among all women with biopsy proven CIN 2/3, 20% were referred due to a LSIL screen.

Cocktail high risk (HR) HPV testing, such as Hybrid Capture 2 (HC2) is positive in 76–80% of LSIL cytology samples [10], [12], [13], too high to be a meaningful positive triage test to efficiently detect precancerous lesions without a high false positive rate. Even as testing has become more refined to identifying specific oncogenic HPV genotypes as an improved positive triage test in cervical cancer screening programs [13], [14], these tests cannot reduce the colposcopic referral rate for those whose HPV infections are highly unlikely to progress to invasive cervical cancer. The addition of a host marker, such as host telomere length, offers the potential to reassure women that they have a very low risk of developing CIN 2/3 prior to their next routine cytologic screen despite a LSIL cytology report. This negative triage test focuses the biomarker on the host rather than the established viral infection.

The purpose of this research is to establish the test characteristics of sensitivity, specificity, positive and negative predictive values of the gain of the chromosome 3q26 region (3q26 gain) for predicting which women with LSIL cytology do not have CIN 2/3 at colposcopy.

## Methods

### Patient Selection

We designed a historical prospective study of women who had presented to the colposcopy clinics at Truman Medical Center, the teaching hospitals of the University of Missouri Kansas City School of Medicine, Kansas City Missouri, USA. To be eligible for inclusion, we reviewed the electronic medical record (EMR) for those women whose index liquid cytology LSIL sample had been archived, who had a colposcopically directed biopsy and who was not pregnant at the time of cytology or biopsy sampling. We were granted ethics approval, and consent waiver, from both the University of Missouri-Kansas City School of Medicine Institutional Review Board (#09-70X) and the Truman Medical Centers’ Privacy Board to use the de-identified data.

### Liquid Cytology Specimens

Clinicians collected the index samples in the SurePath™ system (Tripath, BD, Franklin Lakes, New Jersey, USA) as part of routine cervical surveillance. The specimens were pelleted and resuspended for cytology processing for immediate clinical diagnosis. No other sampling was done on the specimen. The remainder of the resuspended sample was refrigerated at 2–4°C until it was used for 3q26 gain testing. The Bethesda System was used to report the cytology diagnoses (e.g. negative for intraepithelial lesion or malignancy (NILM), atypical squamous cells of undetermined significance (ASCUS), LSIL, high grade squamous intraepithelial lesion (HSIL)). CIN nomenclature was used to report the histologic diagnosis from the biopsy specimen (e.g. CIN grade 1, 2 or 3).

### 3q26 Gain Fluorescence In-situ Hybridization (FISH) Testing

The cytology specimens were mailed at ambient temperature to the Ikonisys laboratory where each was transferred to a 15 mL tube and centrifuged for 6 minutes at 1200 RPM. The supernatant was decanted and the pellets resuspended in 5 mL of KCl for 15 minutes at 37°C. Two milliliters Carnoy’s Fixative was added and the samples centrifuged for 6 minutes at 1200 RPM. The supernatant was discarded and the pellet resuspended in 10 mL of Carnoy’s fixative and incubated overnight. Samples were centrifuged for 6 minutes at 1200 RPM, the supernatant discarded and the pellet resuspended in an appropriate volume of Carnoy’s Fixative. Slides were prepared manually and incubated at 55°C for 10 minutes. Prior to hybridization, slides were immersed in 2X SSC for 2 minutes at 73°C and treated with 1% pepsin for 10–15 minutes at 37°C. Slides were then incubated in 1X PBS for 2 minutes and fixed in 2% formalin for 5 minutes, at room temperature. Slides were washed in 2X SSC for 2 minutes at room temperature and dehydrated in an ethanol series (70%, 85%, 100%).

### Hybridization

The slides were hybridized using the oncoFISH cervical probe kit (Ikonisys, Inc. New Haven, CT). Briefly, 1 µL of 3q26 probe, 1 µL of CEP 7 probe, 1 µL distilled water and 7 µL of hybridization buffer were mixed and applied to the slide. A 22 mm round glass cover slip was placed over the probe and air bubbles were removed. Slides were transferred to a ThermoBrite (Abbot Molecular, Des Plaines, IL), denatured at 76°C for 5 minutes and hybridized overnight at 37°C. Following hybridization, slides were washed in 2X SSC, 0.3% NP-40 at 73°C for 2 minutes then in 2X SSC, 0.1% NP-40 at room temperature for an additional 2 minutes. Slides were counterstained with DAPI (Sigma-Aldrich, St. Louis, MO) for 3–4 minutes at room temperature and dehydrated in an ethanol series. Ten microliters of antifade and a 22×50 mm cover slip were applied and slides were placed at −20°C for at least 15 minutes prior to analysis.

### Slide Scanning

**Figure 1:**
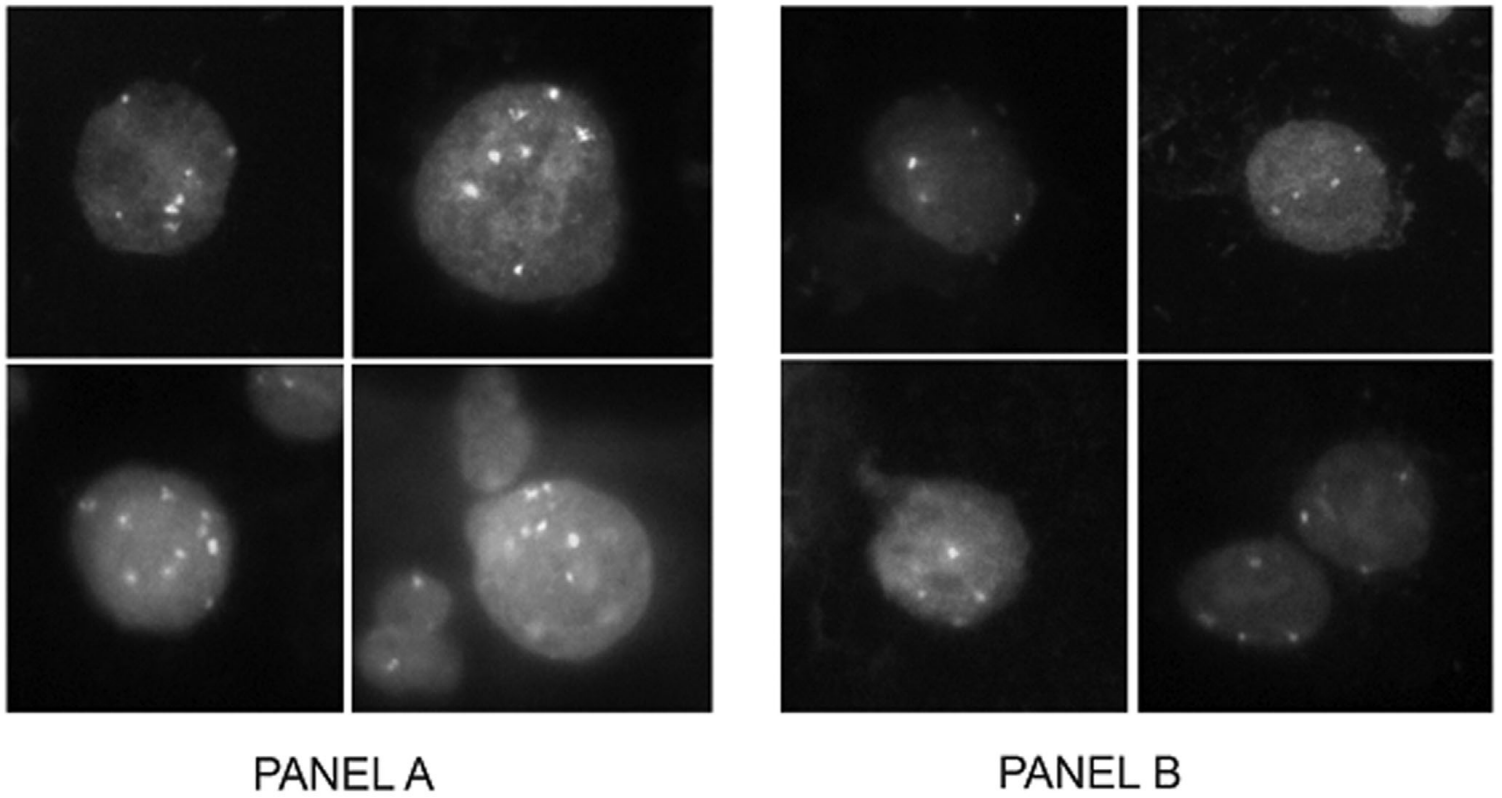
Cells positive and negative for 3q26 gain. Cells positive (Panel A) or negative (Panel B) for 3q26 gain detected in subjects with LSIL cytology. 3q26 fluorescnce in situ hybridization (FISH) signals are colored gold and control centromeric 7 FISH signals are colored aqua. A positive test contains two or more cells exhibiting more than four 3q26 (gold) FISH signals.

We analyzed 65 slides using an automated system developed specifically for rare-cell detection and analysis (Ikonisys, Inc., New Haven, CT). The entire sample was scanned using a 20× objective and both the total number of nuclei and number of nuclei with >2 signals for 3q26 were determined. The nuclei were ordered based on the number of 3q26 FISH signals, and up to 800 nuclei with the highest number of 3q26 FISH signals were imaged using a 40× objective. Nuclei were enumerated for both 3q26 and control centromeric 7 FISH probe signals to determine 3q26 gain. The test was positive if two or more cells with more than four 3q26 FISH signals were detected (Figure 1).

Fourteen cytology samples were unable to be read by automated scanning for the 3q26 gain signal. These slides had less than 50 nuclei imaged at high magnification (40×), and they had an increase in background autofluorescence that could confound the signal to noise ratio. Therefore, following scanning, these fourteen slides were reviewed for 3q26 gain manually, while maintaining blinding to the subjects’ clinical information.

#### Statistics

The power analysis was calculated with Statistica software [15] using a chi square test for one proportion defining significance to include a two-tailed alpha level of 0.05. In order to achieve power of 80% while observing a negative predictive value of at least 85% while assuming a null negative predictive value of 70% we needed a sample of at least 60 subjects. We estimated proportions of sensitivity, specificity and positive and negative predictive values with binomial exact 95% confidence intervals (CIs). Population characteristics of those women whose results were obtained via automation vs. manual review were compared with chi-square testing using a two-sided alpha level of 0.05 as the threshold of significance [15]. The Pearson correlation coefficient was used to determine variable correlations. The results are reported according to the STARD initiative for diagnostic accuracy [16].

## Results

**Figure 2:**
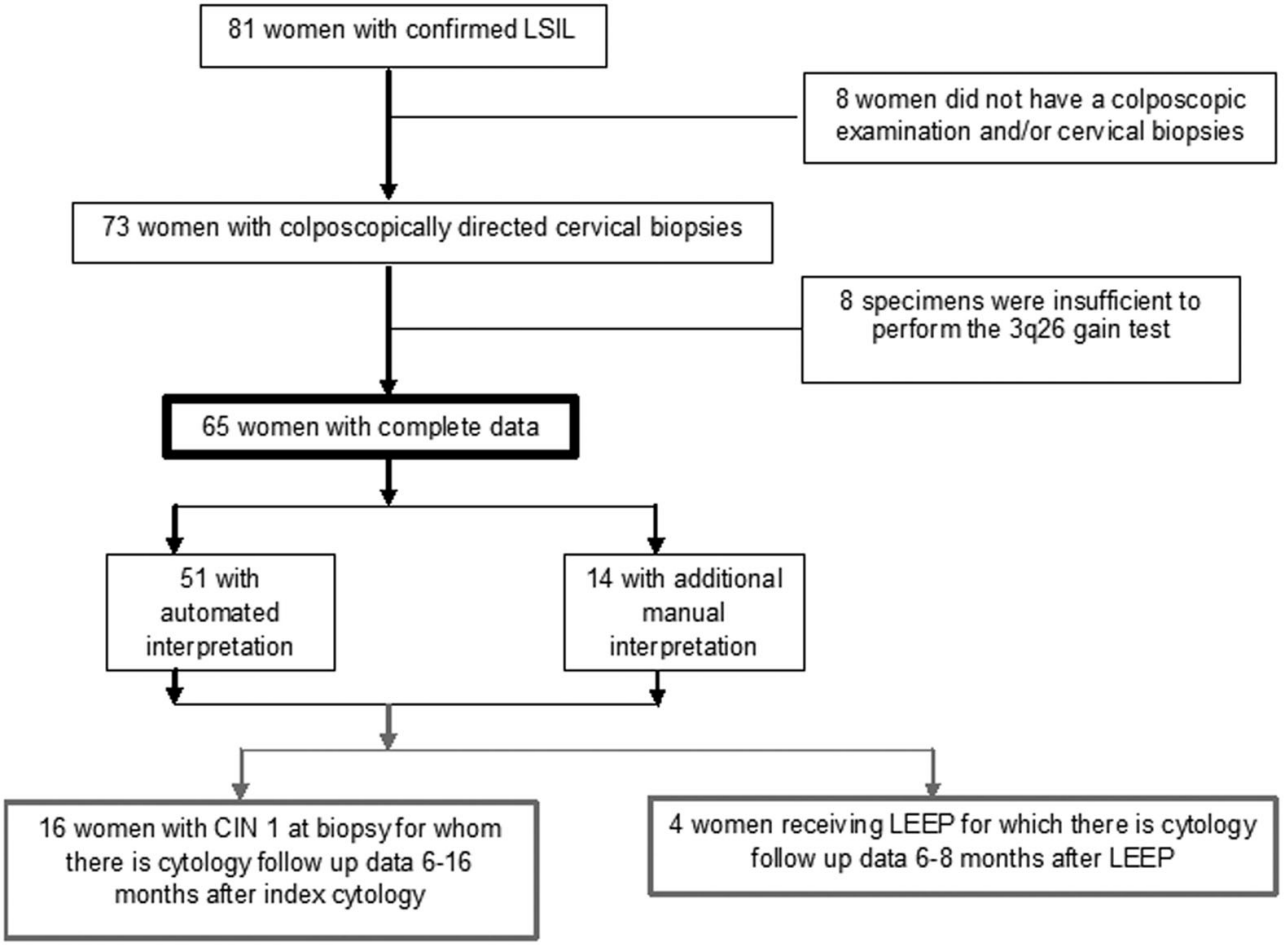
Trial profile.

81 LSIL cervical specimens from unique non-pregnant women were collected between May 2007 and January 2009 and stored at 2–4°C. Of these 81, 73 women had colposcopically directed biopsies on average 36 days from their index cytology (standard deviation (SD): 18). Of these 73 specimens, 65 provided sufficient archived material to run the 3q26 gain test resulting in 65 women with complete index cytology, colposcopically directed biopsy and index 3q26 gain status (Figure 2). Of these 65 women, 16 whose colposcopically directed biopsy was reported as CIN 1 had a repeat cytology sample 6–16 months after biopsy; and four of the 10 women with CIN 2/3 had cytology follow up 6–8 months after their loop electrosurgical excision procedure (LEEP). The 3q26 gain test was performed on average 616 days after collection (SD 157).

The average age of the study population was 32 years (SD 9.6 yrs) ranging from 17–59 years. Most of the women in the study were African-American (51%) or Caucasian (40%). The women had a mean gravidity and parity of 2.6 (SD 1.9) and 2.0 (SD 1.6), respectively, and 40% of the women had had a prior abnormal Pap. Ten of the 65 women (15%) had CIN 2/3 at colposcopy with the remaining having CIN 1 or normal tissue (Table 1).

**Table 1:** Descriptors of LSIL* Subjects.

	N = 65 mean (SD)
Age, years	32 (9.6)
Gravidity	2.6 (1.9)
Parity	2.0 (1.6)
Age of specimen prior to 3q26 gain processing, days	609 (151)
Time from index cytology to colposcopically directed biopsy, days	35 (18)
Race	Number (%)
Caucasian	26 (40)
African American	33 (51)
Hispanic	5 (8)
Other	1 (1)
Prior abnormal Pap history	26 (40)
Histology diagnosis from colposcopic biopsy	
Normal	12 (19)
CIN† 1	43 (66)
CIN 2	6 (9)
CIN 3	4 (6)

* LSIL means low grade squamous intraepithelial lesion.

† CIN means cervical intraepithelial neoplasia grade 1, 2 or 3.

Test characteristics were calculated for 3q26 gain status determined by both automated slide scanning and manual slide review. Seven of the 10 women with CIN 2/3 were positive for 3q26 gain on the index cytology for a sensitivity of 70% (95% CI: 35, 93). The specificity of the 3q26 gain test was 91% (95% CI: 80, 97); the positive predictive value (PPV) was 44% (95% CI: 14, 79) and the negative predictive value (NPV) was 89% (95% CI: 78, 96) (Table 2).

**Table 2:** Test characteristics of 3q26 gain for women with LSIL cytology for detecting CIN 2/3 using automated and manual review of 3q26 gain analysis.

	CIN 2/3	<CIN 2/3	Total
3q26 gain	7	5	12
No 3q26 gain	3	50	53
Total	10	55	65
Sensitivity = 70% (95% CI: 35, 93)			
Specificity = 91% (95% CI: 80, 97)			
PPV = 58% (95% CI: 28, 85)			
NPV = 89% (95% CI: 78, 96)			

PPV means positive predictive value.

NPV means negative predictive value.

CIN 2/3 means cervical intraepithelial neoplasia grade 2 or 3.

<CIN 2/3 means normal or CIN grade 1.

Recalculation of the 3q26 gain test characteristics after exclusion of the manually reviewed slides does not substantially change the four test characteristics of 3q26 gain (Table 3). There was no difference between those slides scanned by automation and those reviewed manually for 3q26 gain in terms of the subjects’ age, gravidity, parity and past history of a prior abnormal Pap test; the age of the cytology specimen prior to processing; or the number of days from cytology sampling to colposcopic biopsy. Similarly there was no correlation between the age of the sample and the 3q26 gain status (r2 = 0.0002, p = 0.92). Compared to the automated scanning, though, the slides manually reviewed occurred less often in Caucasian subjects (14.3% vs. 47.1%, p = .03) and were less likely to be associated with a CIN 1 biopsy (42.9% vs. 72.5%, p = 0.04).

**Table 3:** Test characteristics of 3q26 gain for women with LSIL cytology for detecting CIN 2/3 using automated 3q26 gain analysis alone.

	CIN 2/3	< CIN 2/3	Total
3q26 gain	4	5	9
No 3q26 gain	1	41	42
Total	5	46	51

Sensitivity = 80% (95% CI: 28, 99).

Specificity = 89% (95% CI: 76, 96).

PPV = 44% (95% CI: 14, 79).

NPV = 98% (95% CI: 87, 100).

PPV means positive predictive value.

NPV means negative predictive value.

CIN 2/3 means cervical intraepithelial neoplasia grade 2 or 3.

<CIN 2/3 means normal or CIN grade 1.

Of secondary interest, sixteen women whose colposcopically directed biopsy was CIN 1 returned for repeat cytology 6–16 months (mean 302, SD 96) from their index cytology. None of the women had progressed to HSIL in that time frame for a non-evaluable sensitivity and a 0% positive predictive value. Twelve of the women regressed to normal cytology, one of whom had had a gain in the 3q26 chromosome on her index cytology, representing a false positive 3q26 gain test. None of the four women whose LSIL persisted had a 3q26 gain on her index cytology, making the specificity of the 3q26 gain test for regression or persistence at a 10 month average follow up 94% (95% CI: 70, 100) and the negative predictive value 100% (95% CI: 78, 100).

In addition, four of the women with biopsy confirmed CIN 2/3 had follow up cytology 6 months (mean 203 SD 16) after their LEEP. All LEEP specimens had negative resection margins. All 6 month follow up cytologies were reported as NILM. Two of the women who showed a 3q26 gain at index cytology showed no 3q26 gain at the post-LEEP follow up cytology.

## Discussion

HPV infections create the morphologic effects seen to codify a LSIL entity. Cells must exhibit nuclear enlargement as well as a defined, perinuclear clear area demarcated by a dense cytoplasmic ring (koilocytes); there may be nuclear smudging and binucleation. The nuclear contour of a LSIL cell may be smudged or granular or slightly irregular with hyperchromasia but the chromatin is evenly distributed. The nuclear enlargement seen in LSIL is at least three times that of the normal intermediate cell along with an increased nuclear to cytoplasmic ratio. These changes are most often seen in the superficial and intermediate cells, in sheets or individually [17]. These morphologic changes are irrespective of the oncogenic potential of the HPV infection and do not signify malignant potential by themselves.

While only 2.5% of all women screened for cervical cancer will have LSIL cytology, these women constitute nearly 50% of all the referrals to colposcopy [4], [18], [19]. This high referral rate results from the current management guidelines which acknowledge the inaccuracies of a LSIL cytologic diagnosis and recommend all women be referred to colposcopy because of the fear of missing underlying CIN 2/3 disease [20], [21]. While these conservative guidelines attempt to expedite any precancerous detection, they are costly and may cause psychosocial harm to the majority of women whose LSIL will not indicate a CIN 2/3 lesion before the next screening interval [22], [23].

The natural history of LSIL cytology supports an observational management guideline if the sensitivity and negative predictive value of a triage test is high enough to provide reassurance that the LSIL lesion will not progress to CIN 2/3 before the next screening interval. LSIL lesions caused by oncogenic HPV types regress, on average, in 14 months, ranging from 9–19 months, while those caused by low risk, benign, types regress, on average, in half that time [24]. Progression rates into HSIL from LSIL caused by oncogenic HPV types occur in 12% of women over 18 months, giving a mean time to progression of about six years [24]. For women with HPV 16 associated CIN 1, which constitutes about a quarter of all CIN 1 [25], less than 20% progress to small CIN 3 lesions within 2 years [26] and about 1% progress to less than two quadrants of CIN 3 within 3 years [27].

Positive triage tests to improve the diagnostic accuracy of cytology have included testing for oncogenic HPV types, quantitating viral load, measuring integration and quantifying markers of cell cycle aberration, such as p16ink4a. These triage tests need high specificity and positive predictive value for identifying women whose abnormal cytology hides a true cancer precursor, balanced with an acceptable rate of false positive tests. The results of these positive triage tests would change clinical management from passive surveillance to requiring further diagnostic work up such as referral to colposcopy. Randomized controlled trials show high risk HPV testing, as a triage test after LSIL, to have specificities less than 50% for predicting those women with CIN 2+ disease where CIN 2+ is defined as CIN 2 or 3 or cervical cancer [28]. The specificity of the test decreases as the method of HPV testing changes from HC2 to PCR methods and the endpoint changes from CIN 2+ to CIN 3+ [29]. Viral load performs marginally better with a specificity of 65% for predicting CIN 2+ in women with viral loads ≥100 pg/ml regardless of the initial cytology [30]. The specificity of integration status, depends on the size of the deleted HPV genome fragment for predicting precancerous disease progression; integration status has not been well-operationalized for clinical utility [31]. The specificity of p16 testing in screened women with normal or mild dyskaryosis predicting CIN 2+ disease has been shown to be similar to viral load testing at 68%; while the specificity of HPV 16, 18, 31, 33 and 45 mRNA testing for E6/E7 protein expression was the highest at 75% [32].

A negative triage test would be one whose negative/normal results would modify current aggressive clinical guidelines to allow observation without clinical intervention until the next screening interval without missing a significant portion of diseased women. A negative triage test is one with a high sensitivity for cancer and cancer precursors in addition to a negative predictive value exceeding 97% [33], [34]. For those countries whose clinical guidelines already refer all women with LSIL to colposcopy, a negative triage test could change clinical guidelines to continued passive routine screening surveillance.

A promising new negative triage test for women with LSIL cytology has been the gain of chromosome length of 3q26. Prior studies report greater than 80% sensitivity for the detection of CIN 2/3 from a LSIL population [35]. Chromosome arm 3q contains the human telomerase RNA gene on chromosome band 3q26. Chromosomal telomeres shorten by dropping terminal DNA sequence repeats as the cell repeatedly divides, leading to chromosomal instability. Telomerase is the enzyme that maintains chromosomal length and stability. Overexpression of telomerase, specifically the catalytic subunit of human telomerase reverse transcriptase (TERT), is one of the crucial steps for malignant transformation and cellular immortalization. Gains in chromosome 3q26 commonly represent chromosomal instability in squamous cell cancers and adenocarcinomas, independent of specific TERT overexpression, and are found in a high proportion of head and neck [36], lung [37], esophageal [38], colon [39], pancreatic [40], breast [41], cervix [42] and vulvar cancers [43]. It should be noted that the 3q26 region contains a plethora of loci, including PIK3CA which might also cause cell cycle dysregulation [44].

Prior studies using gains in 3q26 amplification showed increasing proportions of 3q26 gain among women with increasingly more severe cervical cytology and histology fulfilling the biological plausibility and temporal association characteristics needed for this triage test to be clinically relevant in cervical cancer screening [45]–[61]. Recent advances in liquid cytology preparations have facilitated 3q26 gain determination from cytology specimens [62] increasing the likelihood of using this biomarker of genetic instability for negative triage screening. Automation of 3q26 gain evaluation could allow large volume triage of LSIL cytologies [63].

Our results show a sensitivity of 80% and a NPV of 98% for automated scanning for 3q26 gain among women with LSIL cytology at immediate colposcopy; and 100% NPV at the 6–16 month follow up visit after CIN1 biopsy. These excellent negative triage test properties are balanced by reassurance of not missing diseased women as seen from the high specificity of 90% and the PPV of 44%, nearly three times the prevalence rate of CIN 2/3 disease in our study population. These test characteristics are sufficiently high to warrant further prospective investigation of this test as a negative triage tool to change clinical guidelines to conservatively follow LSIL women negative for 3q26 gain instead of the current guidelines of immediate referral to colposcopy.

Other clinical states that may benefit from a host-based negative triage test, such as 3q26 gain, include those women with atypical glandular cells of undetermined significance (AGC) [64], women with vulvar disease [65] and those women with abnormal cytology who also have HIV, organ transplants and other immunosuppressed states [66], [67] who currently are advised to undergo frequent diagnostic work ups.

The limitations of our study include the historical prospective trial design which relies on the integrity of archived liquid cytology material for 3q26 gain testing; and an unknown disease state in women whose LSIL smears were censored from analysis due to incomplete records of a colposcopic endpoint or interval surveillance screens.

A prospective trial in larger numbers of women with LSIL from the general population of wide age ranges is ongoing. This study may determine a more robust characterization of the 3q26 gain test for reassuring women and physicians of the lack of need for immediate colposcopy after a LSIL Pap test.

## References

[1] Sherman ME, Dasgupta A, Schiffman M, Nayar R, Solomon D (2007). The Bethesda Interobserver Reproducibility Study (BIRST): a web-based assessment of the Bethesda 2001 System for classifying cervical cytology. Cancer..

[2] Herbert A, Bergeron C, Wiener H, Schenck U, Klinkhamer P (2007). European guidelines for quality assurance in cervical cancer screening: recommendations for cervical cytology terminology. Cytopathology..

[3] World Health Organization (2003). Eds Fattaneh A. Tavassoéli and Peter Devilee. Pathology and Genetics of Tumours of the Breast and Female Genital Organs.. Lyon: IARC Press.

[4] Eversole GM, Moriarty AT, Schwartz MR, Clayton AC, Souers R (2006). Practices of participants in the college of american pathologists interlaboratory comparison program in cervicovaginal cytology. Arch Pathol Lab Med..

[5] Schiffman M (2007). Integration of human papillomavirus vaccination, cytology, and human papillomavirus testing. Cancer..

[6] Stoler MH, Schiffman M (2001). Interobserver reproducibility of cervical cytologic and histologic interpretations: realistic estimates from the ASCUS-LSIL Triage Study. JAMA..

[7] Solomon D, Schiffman M, Tarone R, ALTS Study group (2001). Comparison of three management strategies for patients with atypical squamous cells of undetermined significance: baseline results from a randomized trial. Journal of the National Cancer Institute..

[8] Kinney WK, Manos MM, Hurley LB, Ransley JE (1998). Where’s the high-grade cervical neoplasia? The importance of minimally abnormal Papanicolaou diagnoses. Obstetrics & Gynecology..

[9] Cox JT, Schiffman M, Solomon D (2003). ASCUS-LSIL Triage Study (ALTS) Group. Prospective follow-up suggests similar risk of subsequent cervical intraepithelial neoplasia grade 2 or 3 among women with cervical intraepithelial neoplasia grade 1 or negative colposcopy and directed biopsy. Am J Obstet & Gynecol..

[10] Kitchener HC, Almonte M, Thomson C, Wheeler P, Sargent A (2009). HPV testing in combination with liquid-based cytology in primary cervical screening (ARTISTIC): a randomised controlled trial. Lancet Oncology..

[11] Arbyn M, Sasieni P, Meijer CJ, Clavel C, Koliopoulos G (2006). Chapter 9: Clinical applications of HPV testing: a summary of meta-analyses. Vaccine..

[12] ASCUS-LSIL Triage Study (ALTS) Group (2003). A randomized trial on the management of low-grade squamous intraepithelial lesion cytology interpretations. Am J Obstet Gynecol..

[13] Arbyn M, Martin-Hirsch P, Buntinx F, Van Ranst M, Paraskevaidis E (2009). Triage of women with equivocal or low-grade cervical cytology results: a meta-analysis of the HPV test positivity rate. J Cell Mol Med..

[14] Rijkaart DC, Berkhof J, van Kemenade FJ, Coupe VM, Hesselink AT (2011). Evaluation of 14 triage strategies for HPV DNA-positive women in population-based cervical screening.. Int J Cancer.

[15] StatSoft Inc (2010). STATISTICA (data analysis software system), version 9.1.. http://www.statsoft.com.

[16] Bossuyt PM, Reitsma JB, Bruns DE, Gatsonis CA, Glasziou PP (2003). Standards for Reporting of Diagnostic Accuracy. Towards complete and accurate reporting of studies of diagnostic accuracy: the STARD initiative. BMJ..

[17] Nayar R, Solomon D (2004). Second edition of ‘The Bethesda System for reporting cervical cytology’ - *Atlas*, website, and Bethesda interobserver reproducibility project. CytoJournal..

[18] Accessed 2012 May 29.. http://www.cancerscreening.nhs.uk.

[19] Cotton S, Sharp L, Little J, Cruickshank M, Seth R (2010). The role of human papillomavirus testing in the management of women with low-grade abnormalities: multicenter randomised controlled trial. BJOG..

[20] Wright TC, Massad LS, Dunton CJ, Spitzer M, Wilkinson EJ (2007). 2006 American Society for Colposcopy and Cervical Pathology-sponsored Consensus Conference. 2006 consensus guidelines for the management of women with abnormal cervical cancer screening tests. Am J Obstet Gynecol..

[21] Jordan J, Arbyn M, Martin-Hirsch P, Schenck U, Baldauf JJ (2008). European guidelines for quality assurance in cervical cancer screening: recommendations for clinical management of abnormal cervical cytology, part 1. Cytopathology..

[22] Hellsten C, Sjostrom K, Lindqvist P (2008). A 2-year follow-up study of anxiety and depression in women referred for colposcopy after an abnormal cervical smear. BJOG..

[23] Le T, Hopkins L, Menard C, Hicks-Boucher W, Lefebvre J (2006). Psychologic morbidities prior to loop electrosurgical excision procedure in the treatment of cervical intraepithelial neoplasia. Int J Gynecol Cancer..

[24] Schlecht NF, Platt RW, Duarte-Franco E, Costa MC, Sobrinho JP (2003). Human papillomavirus infection and time to progression and regression of cervical intraepithelial neoplasia. J Natl Cancer Inst..

[25] Guan P, Howell-Jones R, Li N, Bruni L, de Sanjosé S (2012). Human papillomavirus (HPV) types in 115,789 HPV-positive women: A meta-analysis from cervical infection to cancer.. Int J Cancer.

[26] Insinga RP, Perez G, Wheeler CM, Koutsky LA, Garland SM (2011). Incident cervical HPV infections in young women: transition probabilities for CIN and infection clearance. Cancer Epidemiol Biomarkers Prev..

[27] Nobbenhuis MA, Walboomers JM, Helmerhorst TJ, Rozendaal L, Remmink AJ (1999). Relation of human papillomavirus status to cervical lesions and consequences for cervical-cancer screening: a prospective study. Lancet..

[28] Arbyn M, Martin-Hirsch P, Wentzensen N (2010). Human papillomavirus-based triage of women showing a cervical cytology result of borderline or mild dyskaryosis. BJOG..

[29] Castle PE, Schiffman M, Wheeler CM, Gravitt PE (2010). Impact of improved classification on the association of human papillomavirus with cervical precancer. Am J Epidemiol..

[30] Dalstein V, Riethmuller D, Prétet JL, Le Bail Carval K, Sautière JL (2003). Persistence and load of high-risk HPV are predictors for development of high-grade cervical lesions: a longitudinal French cohort study. Int J Cancer..

[31] Hopman AHN, Smedts F, Dignef W, Ummelen M, Sonke G (2004). Transition of high-grade cervical intraepithelial neoplasia to micro-invasive carcinoma is characterized by integration of HPV 16/18 and numerical chromosome abnormalities. J Pathol..

[32] Szarewski A, Ambroisine L, Cadman L, Austin J, Ho L (2008). Comparison of predictors for high-grade cervical intraepithelial neoplasia in women with abnormal smears. Cancer Epidemiol Biomarkers Prev..

[33] Arbyn M, Ronco G, Cuzick J, Wentzensen N, Castle PE (2009). How to evaluate emerging technologies in cervical cancer screening? Int J Cancer..

[34] Castle PE, Sideri M, Jeronimo J, Solomon D, Schiffman M (2007). Risk assessment to guide the prevention of cervical cancer. Am J Obstet Gynecol..

[35] Jalali GR, Herzog TJ, Dziura B, Walat R, Kilpatrick MW (2010). Amplification of the chromosome 3q26 region shows high negative predictive value for nonmalignant transformation of LSIL cytologic finding. Am J Obstet Gynecol..

[36] Liu Z, Ma H, Wei S, Li G, Sturgis EM (2011). Telomere Length and TERT Functional Polymorphisms are not Associated with Risk of Squamous Cell Carcinoma of the Head and Neck. Cancer Epidemiol Biomarkers Prev.. In press.

[37] Jin G, Yoo SS, Cho S, Jeon HS, Lee WK (2011). Dual roles of a variable number of tandem repeat polymorphism in the TERT gene in lung cancer. Cancer Sci.. http://dx.doi.org/10.1111/j.1349–7006.2010.01782.x.

[38] Hsu CP, Lee LW, Shai SE, Chen CY (2005). Clinical significance of telomerase and its associate genes expression in the maintenance of telomere length in squamous cell carcinoma of the esophagus. World J Gastroenterol..

[39] Safont MJ, Gil M, Sirera R, Jantus-Lewintre E, Sanmartín E (2011). The prognostic value of hTERT expression levels in advanced-stage colorectal cancer patients: a comparison between tissue and serum expression. Clin Transl Oncol..

[40] Suso EM, Dueland S, Rasmussen AM, Vetrhus T, Aamdal S (2011). hTERT mRNA dendritic cell vaccination: complete response in a pancreatic cancer patient associated with response against several hTERT epitopes. Cancer Immunol Immunother..

[41] Zhang B, Beeghly-Fadiel A, Long J, Zheng W (2011). Genetic variants associated with breast-cancer risk: comprehensive research synopsis, meta-analysis, and epidemiological evidence. Lancet Oncol..

[42] Andersson S, Wallin KL, Hellström AC, Morrison LE, Hjerpe A (2006). Frequent gain of the human telomerase gene TERC at 3q26 in cervical adenocarcinomas. Br J Cancer..

[43] Aulmann S, Schleibaum J, Penzel R, Schirmacher P, Gebauer G (2008). Gains of chromosome region 3q26 in intraepithelial neoplasia and invasive squamous cell carcinoma of the vulva are frequent and independent of HPV status. J Clin Pathol..

[44] Ma Y, Wei S, Lin Y (2000). Pik3ca as an oncogene in cervical cancer.. Oncogene.

[45] Li Y, Ye F, Lü WG, Zeng WJ, Wei LH (2010). Detection of human telomerase RNA gene in cervical cancer and precancerous lesions: comparison with cytological and human papillomavirus DNA test findings. Int J Gynecol Cancer..

[46] Kokalj-Vokac N, Kodric T, Erjavec-Skerget A, Zagorac A, Takac I (2009). Screening of TERC gene amplification as an additional genetic diagnostic test in detection of cervical preneoplastic lesions. Cancer Genetics & Cytogenetics..

[47] Alameda F, Espinet B, Corzo C, Munoz R, Bellosillo B (2009). 3q26 (hTERC) gain studied by fluorescence in situ hybridization as a persistence-progression indicator in low-grade squamous intraepithelial lesion cases. Human Pathology..

[48] Sokolova I, Algeciras-Schimnich A, Song M, Sitailo S, Policht F (2007). Chromosomal biomarkers for detection of human papillomavirus associated genomic instability in epithelial cells of cervical cytology specimens. Journal of Molecular Diagnostics..

[49] Tu Z, Zhang A, Wu R, Jiang J, Li Y (2009). Genomic amplification of the human telomerase RNA gene for differential diagnosis of cervical disorders. Cancer Genetics & Cytogenetics..

[50] Umayahara K, Numa F, Suehiro Y, Sakata A, Nawata S (2002). Comparative genomic hybridization detects genetic alterations during early stages of cervical cancer progression. Genes, Chromosomes & Cancer..

[51] Hopman AH, Theelen W, Hommelberg PP, Kamps MA, Herrington CS (2006). Genomic integration of oncogenic HPV and gain of the human telomerase gene TERC at 3q26 are strongly associated events in the progression of uterine cervical dysplasia to invasive cancer. Journal of Pathology..

[52] Heselmeyer-Haddad K, Janz V, Castle PE, Chaudhri N, White N (2003). Detection of genomic amplification of the human telomerase gene (TERC) in cytologic specimens as a genetic test for the diagnosis of cervical dysplasia. American Journal of Pathology..

[53] Heselmeyer-Haddad K, Sommerfeld K, White NM, Chaudhri N, Morrison LE (2005). Genomic amplification of the human telomerase gene (TERC) in pap smears predicts the development of cervical cancer. American Journal of Pathology..

[54] Andersson S, Sowjanya P, Wangsa D, Hjerpe A, Johansson B (2009). Detection of genomic amplification of the human telomerase gene TERC, a potential marker for triage of women with HPV-positive, abnormal Pap smears. American Journal of Pathology..

[55] Cao Y, Bryan TM, Reddel RR (2008). Increased copy number of the TERT and TERC telomerase subunit genes in cancer cells. Cancer Science..

[56] Zhang A, Maner S, Betz R, Angstrom T, Stendahl U (2002). Genetic alterations in cervical carcinomas: frequent low-level amplifications of oncogenes are associated with human papillomavirus infection. International Journal of Cancer..

[57] Wilting SM, Snijders PJ, Meijer GA, Ylstra B, van den Ijssel PR (2006). Increased gene copy numbers at chromosome 20q are frequent in both squamous cell carcinomas and adenocarcinomas of the cervix. Journal of Pathology..

[58] Wilting SM, Steenbergen RD, Tijssen M, van Wieringen WN, Helmerhorst TJ (2009). Chromosomal signatures of a subset of high-grade premalignant cervical lesions closely resemble invasive carcinomas. Cancer Research..

[59] Huang KF, Lee WY, Huang SC, Lin YS, Kang CY (2007). Chromosomal gain of 3q and loss of 11q often associated with nodal metastasis in early stage cervical squamous cell carcinoma. Journal of the Formosan Medical Association..

[60] Heselmeyer K, Macville M, Schrock E, Blegen H, Hellstrom AC (1997). Advanced-stage cervical carcinomas are defined by a recurrent pattern of chromosomal aberrations revealing high genetic instability and a consistent gain of chromosome arm 3q. Genes, Chromosomes & Cancer..

[61] Verri A, Jalali GR, Cecchini G, Diani S, Dorji T (2011). Significant progression of uterine cervical epithelial lesion accompanied by marked increase in 3q26 gene amplification.. LabMedicine.

[62] Caraway NP, Khanna A, Dawlett M, Guo M, Guo N (2008). Gain of the 3q26 region in cervicovaginal liquid-based pap preparations is associated with squamous intraepithelial lesions and squamous cell carcinoma. Gynecologic Oncology..

[63] Seppo A, Jalali GR, Babkowski R, Symiakaki H, Rodolakis A (2009). Gain of 3q26: a genetic marker in low-grade squamous intraepithelial lesions (LSIL) of the uterine cervix. Gynecologic Oncology..

[64] Andersson S, Wallin KL, Hellstrom AC, Morrison LE, Hjerpe A (2006). Frequent gain of the human telomerase gene TERC at 3q26 in cervical adenocarcinomas. British Journal of Cancer..

[65] Aulmann S, Schleibaum J, Penzel R, Schirmacher P, Gebauer G (2008). Gains of chromosome region 3q26 in intraepithelial neoplasia and invasive squamous cell carcinoma of the vulva are frequent and independent of HPV status. J Clin Pathol..

[66] Massad LS, Evans CT, Minkoff H, Watts DH, Strickler HD (2004). Natural history of grade 1 cervical intraepithelial neoplasia in women with human immunodeficiency virus. Obstet Gynecol..

[67] Wong G, Chapman JR, Craig JC (2008). Cancer screening in renal transplant recipients: what is the evidence? Clin J Am Soc Nephrol..

